# Elements of Medicine: A Compendious View of Pathology and Therapeutics; or the History and Treatment of Diseases

**Published:** 1855-11

**Authors:** 


ART. VI.—*Elements of Medicine: a Compendious View of Pathology and Therapeutics; or the History and Treatment of Diseases.* By SAMUEL HENRY DICKSON, M. D., LL.D., Professor of the Institutes and Practice of Physic in the Medical College of the State of South Carolina. Philadelphia: Blanchard & Lea. 1855.

We have often expressed the opinion that a good work on general practice was a desideratum which amounts to an impossibility. Since the publication of Wood's Practice on this side, and of Watson's on the eastern shore of the Atlantic, we believe that no writer has attempted the task.

Premising that Dr. Dickson is a well known and experienced teacher, that his opinion on practical matters bears with it great weight at the south, and that he is probably as competent as any man to write a useful work on general practice, we must still be permitted to doubt even his capacity to force into seven hundred and fifty octavo pages any sufficient statement of the history and treatment of the long catalogue of diseases recorded in his index.

The arguments in favor of works of the present intention and scope, are that allowance must be made for the limited time and purse of the student or practitioner, that we should not expect too high a scholarship in books adapted to a profession largely made up of uneducated men, and that our medical writings must be adapted to our present brief course of study.

As it is impossible for us to pass through the subjects treated by Dr. Dickson, *seriatim*, and moreover as this is the first time that it has been our duty to review a work of this sort, we propose to discuss rather the merits of the class than the book itself, and to use Dr. D.'s "Practice" only in illustration of our general argument.

Are we right in assuming that allowance should be made for the limited time and purse of the student? We think not, for in doing so we concede that the profession is one which will not pay for the study required to practice it well, and as a consequence that it must remain ill-paid and ignorant.

It is evident that the question involves great difficulties, but we are very unwilling to impale ourselves on that horn of the dilemma thus briefly stated.

What is worth doing at all is worth doing well. A person assuming to practice medicine is not justified in saying to himself that he will only attain to the same degree of excellence as that possessed by Dr. A. or B. He is bound to give the *best* efforts of his mind, and to *excel* Dr. A. or B., if Providence has given him the necessary brains. Neither is he justified in saying to himself that as he is ill-paid he cannot afford to purchase books, and that he can only be required to furnish good skill when he is paid for good skill. If the profession will not support talent, it should not support ignorance, and the only honest alternative is to abandon it. There is no law of ethics requiring a man to practice physic against his conscience.

We presume that no one will seriously dispute the logic of these assertions, and we shall take no pains to prove them. But if we admit them, a logical necessity at once arises, and it is evident that the literature of the profession should be adapted to the most advanced stage of excellence. Nor does this view carry us beyond an entirely rational and practical result. The knowledge required for the highest excellence in the practice of medicine is within the reach of fair talent. It need not be required that the practitioner should be a Faraday in chemistry, but he should be competent to a qualitative analysis of the excretions. He may be privileged to ignorance of the teleosaurus, and of that transcendental vertebra 'yclept the streptospondylus, provided he can avoid the anomalous obturator in operating for femoral hernia, or knows enough of cell-life to rectify a disordered nutrition.

The attainment which is thus demanded can never be reached if the practitioner confines his reading to Bell and Stokes, Watson, Wood, or Dickson. The very reasoning on which these learned writers base their practical conclusions is an unknown tongue to him, and the premises being deficient, a blunder in their application follows as a matter of course.

Another phase of the same fault in works on general practice, is the attempt to condense a page into a sentence. Turning to page 601, article "Bronchitis," of the work which, if not under review, serves as a text for our remarks, and we find the following—comprising all that is said of the diagnosis of chronic bronchitis:

"*Diagnosis.* The distinction between chronic bronchitis and tubercular phthisis is often difficult. In the latter, there is less crepitus or râle, less soreness of the trachea and thorax, more tendency generally to hæmoptysis and less expectoration in the early stages. In their advanced stages, we can draw no line between them, except from their previous history."

Suppose a young practitioner with this book as the only one on practice in his library, what a marvellously intelligent diagnosis he would make! and how comfortably he would rest his conscience on Dr. Dickson's assertion that a correct diagnosis is impossible in the advanced stages!

As equally illustrative of this inherent vice in "works on practice," we give *entire* his account of the physical signs in tuberculosis:

"The *physical signs* of the successive stages of phthisis have been carefully studied. In the incipient condition they are negative rather than positive. (?) Tubercles are situated chiefly at the apex of the lung at first; there may *(will)* be dullness just under the clavicle on percussion. When softening occurs, the surrounding inflammation consolidates the tissue, and we have loss of vesicular murmur and marked dullness; *(never before?)* these spread with the spreading infiltration and deposition. *(Rather loose description.)* When a vomica is formed, and a cavity more or less emptied, we have resonance and pectoriloquy; to which are added the metallic tinkling, (*perhaps*) and when the cavity grows large, amphoric resonance. Ulceration may perforate the pleura, when we shall have pneumo-thorax."

And this is all that is said of the physical signs of that disease, upon the early diagnosis of which so many lives are at this moment depending!

We must go back to our starting point. Dr. Dickson is a scholar and competent to teach, but not in the strait jacket of a work on general practice. Is he himself willing that his capacity to diagnosticate tubercle should be judged by his own words as we have given them? Believing as we do that the highest order of talent must fail in an attempt like this, that the multiplication of such books must deteriorate the standard of medical learning, we trust that, as in our short career as a critic this is the first, it may also be the last volume on the "Practice of Medicine" we shall have occasion to review.

